# Texting While Driving: A Literature Review on Driving Simulator Studies

**DOI:** 10.3390/ijerph20054354

**Published:** 2023-02-28

**Authors:** Gheorghe-Daniel Voinea, Răzvan Gabriel Boboc, Ioana-Diana Buzdugan, Csaba Antonya, George Yannis

**Affiliations:** 1 Department of Automotive and Transport Engineering, Transilvania University of Brașov, 29 Eroilor Blvd., 500036 Brasov, Romania; 2 Department of Transportation Planning and Engineering, National Technical University of Athens, 5 Heroon Polytechniou str., GR-15773 Athens, Greece

**Keywords:** texting while driving, distracted driving, simulator study, literature review

## Abstract

Road safety is increasingly threatened by distracted driving. Studies have shown that there is a significantly increased risk for a driver of being involved in a car crash due to visual distractions (not watching the road), manual distractions (hands are off the wheel for other non-driving activities), and cognitive and acoustic distractions (the driver is not focused on the driving task). Driving simulators (DSs) are powerful tools for identifying drivers’ responses to different distracting factors in a safe manner. This paper aims to systematically review simulator-based studies to investigate what types of distractions are introduced when using the phone for texting while driving (TWD), what hardware and measures are used to analyze distraction, and what the impact of using mobile devices to read and write messages while driving is on driving performance. The review followed the Preferred Reporting Items for Systematic Reviews and Meta-Analysis extension for Scoping Reviews (PRISMA-ScR) guidelines. A total of 7151 studies were identified in the database search, of which 67 were included in the review, and they were analyzed in order to respond to four research questions. The main findings revealed that TWD distraction has negative effects on driving performance, affecting drivers’ divided attention and concentration, which can lead to potentially life-threatening traffic events. We also provide several recommendations for driving simulators that can ensure high reliability and validity for experiments. This review can serve as a basis for regulators and interested parties to propose restrictions related to using mobile phones in a vehicle and improve road safety.

## 1. Introduction

Road safety is increasingly threatened by distracted driving. One of the highest-risk forms of distracted driving is texting while driving (TWD) [1,2] alongside talking on the phone while driving (TPWD) [3,4]. After decades of research, the statistics show that the risks associated with TWD are very high [5]. According to the United Nations Road Safety statistical data [6], car traffic crashes cause more than 1.35 million deaths and injure as many as 50 million people annually worldwide, and a significant cause of such crashes is distracted driving [7]. Considering that, distracted driving has become a common topic in studies that aim to find solutions to reduce traffic injuries and death.

A general approach to road safety is to identify and analyze all distraction activities that can lead to a crash [8,9]. For example, in 2019, the road traffic injuries statistics showed that a total of 36,096 deaths were reported in the US, of which 8.7 percent were attributed to driver distraction due to phone use, eating, and so on [10]. In the EU, the European Commission reported a decrease in the number of fatal crashes in 2020 compared to 2019 by up to 17%, a year in which it was estimated that 18,800 people lost their lives in car crashes [11]. Lower traffic due to the pandemic restrictions during the COVID-19 pandemic had a clear, though unmeasurable, contribution to this. Although the average number of fatalities has decreased (for example, Romania showed a decrease of 12%), some countries reported an increase (Switzerland reported an increase of 21%) [12], which indicates that there is still a need for more countermeasures. Romania, on the other hand, is at the top of the list when it comes to road traffic fatalities, with 85 car crashes per million inhabitants [13]. These crashes are caused by distraction factors, both internal (e.g., a smartphone) and external (e.g., a roadside advertisement), in addition to situations in which the driver has consumed alcohol or prohibited substances [10]. 

Road safety could be improved if it is analyzed from several perspectives. For example, a bibliometric review covering 10 years of research focused on cyclist safety has proposed several recommendations that can lead to well-designed and safer bike networks [14]. In [15], the authors investigated the effect of cardiovascular and respiratory physiological parameters on driver’s mental workload. The findings are conflicting, with some studies suggesting that variations in heart rate (HR) and heart-rate variability (HRV) can reflect changes in mental workload. Due to external influences, respiratory rate (RR) demonstrated little importance in most studies, and it has not been a popular choice for researching driving mental workload. The authors conclude that machine learning algorithms combined with subjective and objective data may yield accurate results in assessing mental effort.

Driver distraction can be defined as “any activity that diverts attention from driving, including talking or texting on the cell phone, eating and drinking, talking to people in the vehicle, fiddling with the stereo, entertainment or navigation system” [16]. The most common sources of distractions are mobile phone use, interaction with passengers, drinking, eating, and controlling in-vehicle devices [9]. There are three basic techniques to determine the distracted state of the driver: studying drivers’ visual scanning patterns, detecting physiological signals, and evaluating driving performance. Driver distraction is often studied and analyzed using various equipment, such as driving simulators, eye-tracking devices, and so on [17,18,19,20]. Most of the studies demonstrated that a driver’s performance could be influenced when a non-driving secondary task is performed at the same time while driving (e.g., cell phone use, TWD, etc.). Therefore, many governments, including those in Europe, the United States, and other countries across the world, have approved restrictions on cell-phone use while driving [21,22,23].

According to [24], driving performance is defined as “performance of the driving task”, where the driving task includes “all aspects involved in mastering a vehicle to achieve a certain goal (e.g., reach a destination), including tracking, regulating, monitoring and targeting”. The driving task requires a wide range of cognitive and physical abilities, such as perception, attention, decision-making, and situational awareness [25]. Thus, driving performance is a crucial indicator of a driver’s ability to operate a vehicle safely and effectively. To comprehensively assess a driver’s capabilities while driving, it is essential to analyze all relevant driving performance parameters, such as lateral control through the standard deviation of lateral position [26], lateral clearance and time-to-danger [27], longitudinal control, reaction time, gap acceptance, eye movement, and workload measures [28]. However, drivers might get so distracted by an activity or event that they cannot react promptly, thus compromising their ability to drive safely. Different types of distractions can influence driving performance, such as visual (the driver is not looking at the road), manual (one hand or both hands are off the steering wheel, e.g., text messaging), and cognitive (the driver is not mentally present while driving, as the attention is focused on the secondary task, e.g., focus on phone) [29]. For example, initiating, writing, and sending a text message while driving involves visual, manual, and cognitive resources. The main effects of distracted driving are increased steering-wheel deviations [30], higher standard deviations of lateral lane position [17], increased reaction time [18,31], lower longitudinal control [32], increased brake time [33], and decreased driving speed [34]. 

In recent years, several smart devices that are worn or attached to the body have been developed that have hands-free functions and can stay connected to the network at any time. Wearables frequently utilize various input modalities (such as touch, speech, or gesture), making their functionalities even more accessible to drivers on the road than a cell phone. Several studies have concluded that the use of mobile or portable devices while driving, such as smartwatches, navigation systems, and Google Glass, has been found to pose a risk to driving safety comparable to conversing on a mobile phone [35]. For example, Glass-delivered messages did not eliminate the distracting cognitive demands, finding that both Google Glass and writing a message on the phone require the same attention resources. Moreover, whether it comes from a smartwatch or smartphone, engaging with notifications carries the risk of taking the attention from the driving task [36]. 

Many researchers have used driving simulators to collect data that can improve road safety, identify and analyze driving profiles, and propose recommendations or policies. Experiments employed in a secure, versatile, and controlled environment have allowed scholars to study potentially dangerous driving scenarios and infer valuable knowledge. However, some possible drawbacks should be mentioned, mainly the external validity (the degree to which a real-world environment can be replicated), the high initial acquisition cost, and the simulator sickness which may be experienced by novice participants [37,38]. 

Research driving simulators in the early eighties, such as HYSIM—Highway Driving Simulator [39], consisted mainly of a fixed-based platform and an interactive visual–audio application. The main improvements that followed were increased graphics quality, advanced motion representation through Stewart motion platforms (Six Degrees of Freedom, 6DOF), cabin and control equipment, realistic vehicle sounds, and environmental factors [40]. Driving simulators were typically described using a three-level system (low-level, mid-level, and high-level) but without having a specific classification criterion [37]. Other classifications were proposed by [41] (Levels 1, 2, 3, and 4; however, the criteria are not explicitly defined), [42] (their approach included a five-band classification with six main parameters), and [37] (A, B, C, and D levels; the criteria were adapted from Helicopter Flight Simulation Classification and include four sets of parameters: general, motion system, visual system, and sound system). The papers included in this work were classified according to [37] because of their explicit and well-defined methodology. 

High-level driving simulators can offer some advantages, such as increased awareness of the surrounding environment due to high-resolution and wide field-of-view display systems [43]. Low-level driving simulators also have well-documented benefits, such as decreased simulator sickness and increased portability and affordability. The work of [44] highlighted the issue of visual fidelity and proposed a methodology to design, calibrate, and use driving simulators. Moreover, [45] showed that visual fidelity significantly impacts driving performance. Based on the acquired knowledge from the current work, we propose several recommendations for driving simulators that can ensure high reliability and validity of the experiments. 

This review aims to highlight the impact of using mobile devices to read and write messages while driving in a simulated environment, with the overarching goal of enhancing traffic safety through several recommendations and pointing out future research directions. The paper’s content focuses on four research questions (RQs) that emphasize the general characteristics that contribute to the need of improving traffic safety: 

RQ1: What types of distractions are introduced when using the phone for TWD?

RQ2: What types of hardware devices were used during experiments to analyze the driver’s performance? 

RQ3: What measures were used to predict and analyze distractions?

RQ4: What is the impact of using mobile devices to read and write messages while driving?

The overall structure of the paper is as follows: Section 2 describes the research methodology. Section 3 presents the results, with a focus on answering to the RQs mentioned above. Section 4 presents the main findings, the proposed recommendations for future research, and the limitations of the work. Finally, Section 5 draws the conclusions of this review of the literature.

## 2. Method

The review was conducted by following the Preferred Reporting Items for Systematic Reviews and Meta-Analyses extension for Scoping Review (PRISMA-ScR). Scoping reviews aim to determine the scope or coverage of a body of the literature on a given topic [46] and identify key concepts and types and sources of evidence to inform practice, policymaking, and research [47]. For this review, we followed the checklist given in [48]. 

### 2.1. Protocol

The manuscript was not previously recorded on PROSPERO or published before, even if the protocol was written before the work began. 

### 2.2. Eligibility Criteria and Study Selection

The studies that met the following criteria were included in the review: full-text, original research in a peer-reviewed journal, published in the English language, and included driving simulators. There was no restriction on the publication year. 

Studies were excluded from the review according to the following criteria: commentary manuscripts; reviews of the literature; editorials; short papers; magazines; dissertations; book chapters; conference papers; non-academic publications; papers that are not available in full text; and studies irrelevant to the research, i.e., that did not investigate the relationship between distracted drivers, mobile phone, use and driving simulators.

We preferred to include only journal articles in our review to maintain high scientific relevance, as they are subject to rigorous review, unlike other types of publications, including conference articles. 

### 2.3. Information Sources 

The following databases were searched in three phases (on 08 January 2021, 10 May 2021, and 14 November 2022): ISI Web of Knowledge, Scopus, Science Direct, SAGE Journals, and ProQuest. 

### 2.4. Search

The review of the literature was conducted with a combination of keywords: “distraction”, “phone”, and “driving simulator”. Additional terms were identified during the first investigation and were used in combination in the search process: “distracted”, “disruptive”, “smartphone”, “mobile phone”, “cell phone”, and “simulation”. Example of search strategy for Scopus database:

ALL ((“distracted” OR “disruptive” OR “disturbing” OR “distraction”) AND (“driving” OR “driver” OR “driver behaviour”) AND (“car” OR “vehicle” OR “automobile” OR “truck”) AND (“simulator” OR “simulation” OR “virtual environment” OR “simulated environment”)) AND (LIMIT-TO (DOCTYPE, “ar”)).

As can be seen, no limit was imposed for the year of publication.

### 2.5. Study Selection

The five abovementioned electronic databases were searched, and the title, abstracts, and other details were downloaded to EndNote (version X9, Clarivate, Philadelphia, PA, USA) for screening. In the first phase, they were screened only by the title and abstract, and after removing the irrelevant articles, the full-text documents of the remaining ones were uploaded in EndNote for the second screening phase. Screening and selection were performed independently by two of the authors (RGB and GDV) and were validated by the third author (CA). Disagreements were resolved through consensus. 

**Figure 1:**
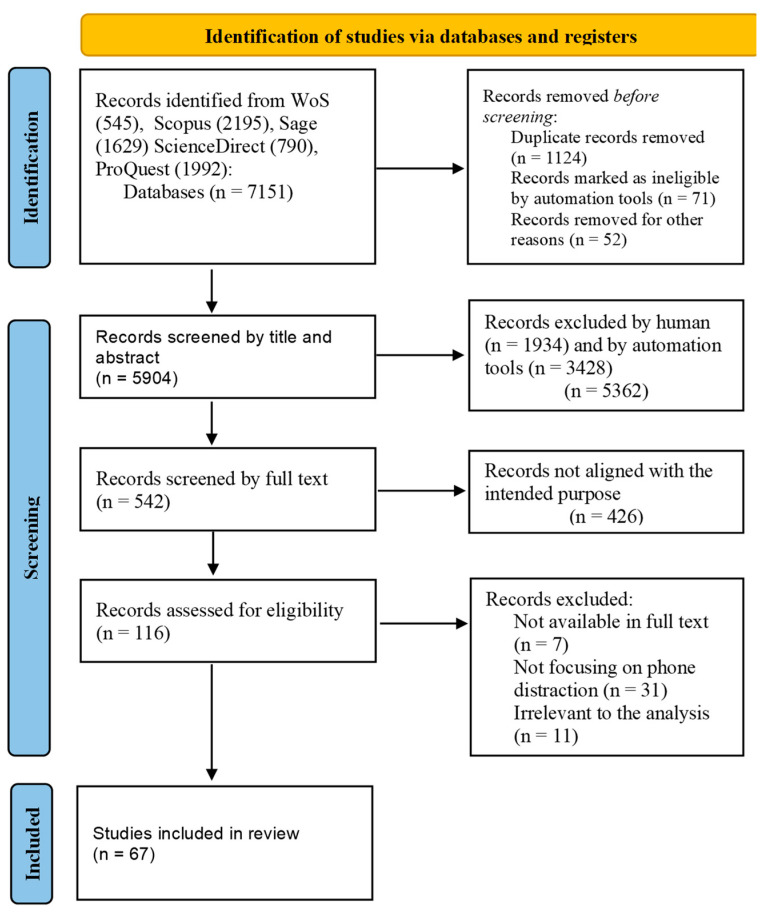
Study identification and selection based on the PRISMA-ScR flow diagram.

The search strategy is shown in Figure 1. Through this selection procedure, 7151 papers were obtained. After removing the duplicated ones, this number was reduced to 5904 papers. Titles and abstracts were analyzed, and articles were included in the review if they were related to studies that investigated the use of mobile phones while driving in a simulator. A total of 542 articles were found, but 475 of them were excluded due to the following reasons: some of them were conference articles, some did not use a car simulator, others were not available for download or were review articles, some assessed pedestrian distraction or the car’s navigation system, others did not use the telephone as a distraction factor, 1 was scholarly paper, 1 used listening audiobooks as a distraction factor, 1 was about e-hailing, and 2 were duplicated. In addition, this paper is intended to be a second part of the work [3], in which the distraction caused by talking on the phone was taken into account. In this regard, the papers focused on talking on the phone were excluded. However, the articles that dealt with the evaluation of both activities—talking and texting—were not removed. Finally, 67 articles were selected for data extraction in this systematic review of the literature. 

### 2.6. Data Extraction

As previously mentioned, the data extraction was performed by two authors (RGB and GDV) and was then validated by a third author (CA). A Microsoft Excel spreadsheet was created to centralize the following information: first author, year of publication, journal name, region (the country where the experiment took place), institution where the research was conducted, sample size, age, gender, and driving experience, type of simulator, driving scenario, tracking device, type of distraction factors, distraction task, type of evaluated measures, effect on a performance measure, independent variables, and statistical analysis technique.

Each reference was read in its entirety by the designated author, and the extracted data were added to the table. The location was based on the country from where the participants were recruited. If the user study involved samples from different countries, we considered the institution’s location that managed the experiment.

The extracted information was classified into 4 categories related to the characteristics of the studies and the four research questions: “What types of distractions are introduced when using the phone for TWD?”, “What types of hardware devices were used during experiments to analyze the driver’s performance?”, ”What measures were used to predict and analyze distraction?”, and “What is the impact of using mobile devices to read and write messages while driving?”.

### 2.7. Synthesis of the Results

The results of the literature review are given in the following section, with each subsection corresponding to an objective or a research question proposed in this study.

## 3. Results

### 3.1. Characteristics of Studies

The main characteristics of the papers, such as publication date and demographic data, are briefly presented in Appendix A Table A1. The 67 studies selected for the review cover a range of 21 years (2002–2022). The number of published papers varies, from 1 paper in 2002 and 2003 to 10 papers in 2021. The highest number of articles were published in 2021. The studies included in the review were published in the following journals: *Transportation Research Part F: Traffic Psychology and Behaviour* (*n* = 13); *Accidents Analysis and Prevention* (*n* = 12); *Applied Ergonomics* (*n* = 4); *Transportation Research Record* (*n* = 4); *Human Factors* (*n* = 3); *Traffic Injury Prevention* (*n* = 3); and several other journals, such as *Safety Science*, *IEEE Access*, *Journal of Safety Research*, and *Transportation Research Part C: Emerging Technologies*.

**Figure 2:**
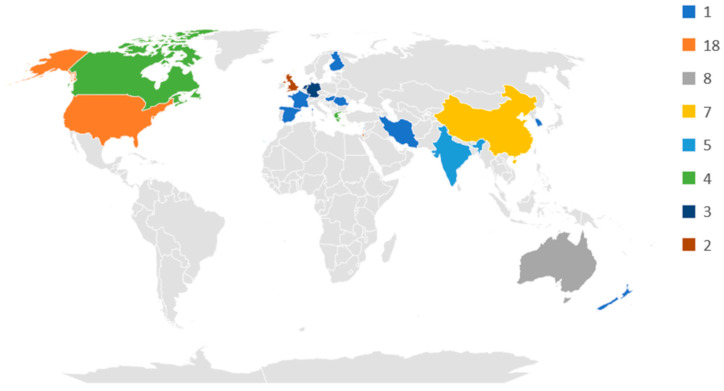
Distribution of papers by country/region.

Most of the studies were developed in North America (*n* = 22), and more particularly in the USA (*n* = 18) (Figure 2). The other studies were conducted in Europe (*n* = 19), Asia (*n* = 17), and Oceania (*n* = 9). In Europe, most publications are from Greece (*n* = 4), Germany (*n* = 3), and The Netherlands (*n* = 3). In Asia, most of the publications are from China (*n* = 7) and India (*n* = 5), and from Oceania, most studies were developed in Australia (*n* = 8).

**Figure 3:**
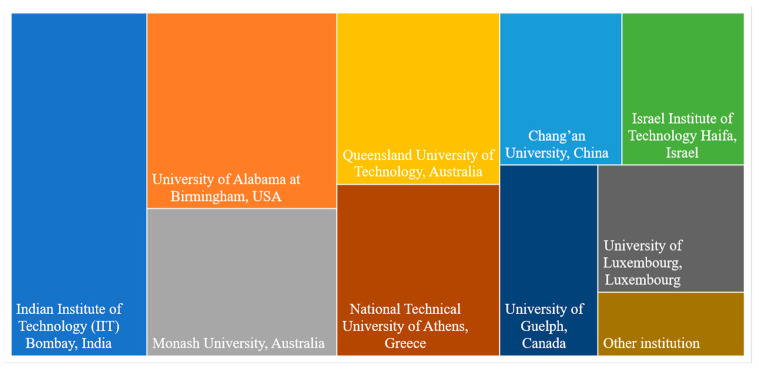
Distribution of papers by research institution.

American, Indian, and Australian research institutions dominate the total number of articles focused on assessing the impact of phone use while driving in virtual environments (Figure 3). Most studies were developed at the Indian Institute of Technology (IIT) Bombay (*n* = 5), followed by the University of Alabama at Birmingham (*n* = 4), Monash University (*n* = 3), and Queensland University of Technology (*n* = 3).

**Figure 4:**
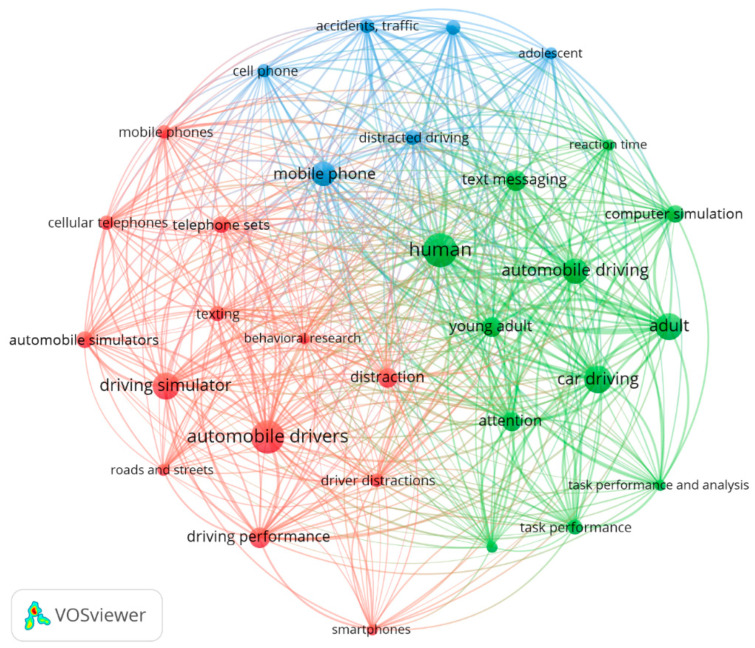
Network diagram of the most frequently used terms.

The analysis of co-occurrence terms was performed using VOS Viewer software version 1.6.18 in order to identify the most frequently used terms and the relationship between them. The minimum number of occurrences of a keyword was selected to be 10, resulting in 35 terms that meet the threshold of the total of 716 keywords. The result of the co-occurrence analysis is presented in Figure 4. As can be observed, the most frequently used keyword was “human”, with 31 occurrences, followed by “automobile drivers”, “car driving”, “driving simulator”, and “mobile phone”. The co-occurrence network map generated by VOS Viewer suggested the division be into three clusters differentiated by colors.

**Figure 5:**
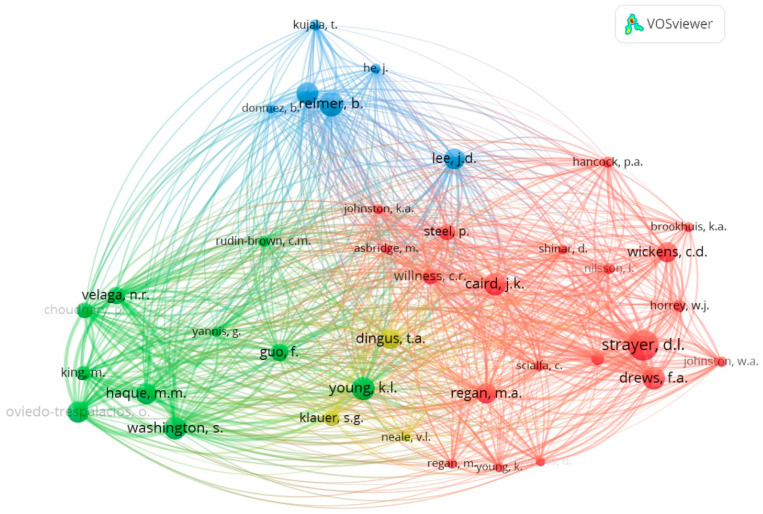
Author co-citations network.

In order to infer connections between the authors and their research topics, the co-citation network was also examined using VOS Viewer. This network entails recognizing pairs of authors who were referenced together in the same publications. Figure 5 shows the results in which the minimum number of citations of an author was set to 20. A number of 39 authors meet the threshold, and four clusters are distinguished.

The selected studies included a sample of 3033 participants (*n* =1984 male; *n* = 1049 female) who participated in simulated driving experiments. The minimum number was 14 [49], and the maximum was 134 [50] participants per study. The gender distribution was not mentioned in two of the extracted studies. 

The age of the participants is between 16 and 79 years old; however, in 17 studies, the age interval is not reported. However, the mean age is reported in more studies (*n* = 59), and the unweighted mean age is 39.6 years across all of these studies. Moreover, the standard deviation is mentioned in 52 studies and is 4.98 across all studies. Only two articles do not mention the age range, the mean age, and the standard deviation.

All participants were assumed to be clinically healthy, except for the participants in one study focusing on teens with and without ADHD [51].

### 3.2. RQ1: What Types of Distractions Are Introduced When Using the Phone for TWD

**Figure 6:**
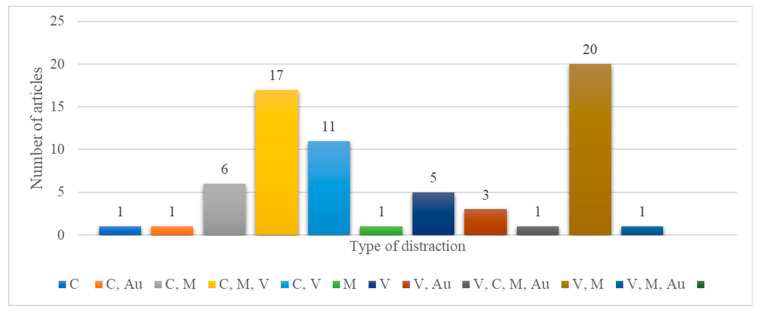
Distribution of papers by the source of distraction type (V—visual; Au—auditory; M—manual (physical); and C—cognitive).

To find out what sources of distraction were used in the studies, we extracted the information on the type of distraction and divided the distractions into the following categories according to [52,53]: visual (V), auditory (Au), manual (M) (physical), and cognitive (C) distraction. The results are presented in Figure 6, as well as in Appendix A Table A1 for each individual study. As can be seen, most articles (34% of the total number of papers, *n* = 23) considered both manual and visual components when assessing the effects of performing secondary tasks while driving. Each secondary task contains one or more components. Examples of visual distractions include interaction with in-vehicle devices [54], the use of smartphone applications while driving [55], looking around, and so on. Auditory distractions emerge when drivers focus on other sounds, such as the ringing of the phone, voice conversations, the radio, etc. Manual distractions involve eating [56], drinking [29] while driving, or doing anything other than manipulating the steering wheel. Finally, cognitive distractions occur when the driver has his/her mind in another place and fails to see what is important on the road. Studies showed that TWD could introduce all of these types of distractions, and even for short durations, they might lead to driving errors and even crashes [57]. Furthermore, most activities unrelated to the driving task combine these four modes [58]. For instance, the most common compound distraction is a visual–manual distraction, defined as a secondary activity that involves using hand gestures to manipulate a visual interface [59]. 

While some articles focused on the visual component [55,60,61], others considered two, three, or even four types of distractions. For instance, both cognitive and visual components were highlighted in [29,62,63]; cognitive and manual components were presented in [64,65]; and visual–manual distraction was evaluated in [35,66,67]. As we have seen, only one article considered all four components of distraction: [68]. In this paper, visual–manual and auditory–vocal interfaces were evaluated, but also the subjective workload was considered as a measure of cognitive distraction.

Some studies investigated the effects of cell phone use in comparison with other secondary tasks, such as talking to a passenger (two studies: [49,69]), eating (four studies: [56,57,70,71]), radio tuning (five studies: [67,69,72,73,74]), using navigation systems (three studies: [33,58,74]), taking pictures [75] or selfies [76], adjusting climate control [72], reading emails (three studies: [55,63,77]), drinking [29], watching video and using social media [63], switching display view and searching songs [55], and sharing numbers [76]. Other studies compare phone use with other types of devices, such as the smartwatch (three studies: [36,68,78]) and Google Glass (two studies: [54,79]). Moreover, instead of using the phone for texting, some researchers used smartphones to perform tasks on social media, such as using Facebook (three studies: [20,80,81]), Snapchat, Instagram [82], Whatsapp [83], or some self-developed applications [60,84]. In one study, the use of mobile phones while driving was evaluated in parallel with drunk driving: [85].

The distraction tasks were divided into two categories: handheld (HH)—holding the device in hand; or hands-free (HF)—performing the task without using hands to hold the device. In 86% of the studies (*n* = 51), the task was performed using HH devices. In 5 studies, both HH and HF devices were used, and in 11 studies, the HF devices were preferred. 

### 3.3. RQ2: What Types of Hardware Devices Were Used during Experiments to Analyze the Driver’s Performance?

#### 3.3.1. Driving Simulator Equipment

Regarding the simulators used in the analyzed studies, 84% of experiments (*n* = 56 studies) were conducted in fixed-based simulators. The other experiments were carried out in driving simulators equipped with motion systems having from 2 to 6 degrees of freedom (DOFs). Each study was classified according to the work of [40], which proposed a classification method for driving simulators that was adapted from flight-simulator classification standards (see Appendix A Table A1). The proposed classes were defined by taking into consideration four sets of criteria: general information, such as environmental modeling and the hardware complexity of the replicated vehicle; the presence of a motion system and the number of degrees of freedom; visual capabilities, especially the field of view; and the sound system which is essential for driver immersion. Class A simulators are at the bottom of the list with no requirement for the motion platform, basic cabin equipment, and basic visual and sound capabilities. Custom-made driving simulators in class A include a desktop computer, steering wheel, gas pedal, and brake pedal, as in the following works: [61,67,86,87]. On the other end, class D simulators require a motion platform with a minimum of six DOFs, at least 180 degrees field of view, and a realistic visual and acoustic environment. Class B simulators were the most popular, as they were used in 36 studies, followed by class A, with 21 studies; class C, with 4 studies; and last but not least, class D simulators, with 6 studies. 

The following class C and D simulators were identified: CARRS-Q Advanced Driving Simulator [76,88,89], the moving-base driving simulator from Würzburg Institute for Traffic Sciences [63], DS-600c Advanced Research Simulator developed by DriveSafety (3 studies: [20,73,82]), Ford’s VIRtual Test Track EXperiment [72], and VS500M driving simulator [30]. One experiment was performed in a driving simulator with three DOFs: [90], and three experiments were performed in two-DOF driving simulators: [20,55,82]. We also extracted some commercially available class A and B driving simulators: Foerst Driving Simulator (three studies: [81,91,92]), PatrolSim high-fidelity driving simulator [66], NADS MiniSim [36], and EF-X from ECA-Faros (two studies: [31,80]). Most systems are developed by Systems Technology Inc., Hawthorne, CA, USA, both hardware and software (used in 10 of the included articles).

The type of display varies among the studies between screen-based projection systems and systems containing monitors. Thirty-nine studies used monitors, ranging from a single monitor to a system of five monitors, and twenty-seven studies in which the display system was based on projectors. The number of screens on which the images were projected ranged from 1 to 7. One paper did not clearly report the information related to the display. The visual field of view (FOV) varied between 40° and 300° for horizontal view and between 24° and 60° for vertical view. However, this information is not reported in a large number of articles (over 16). The most advanced display is installed on the DS-600c advanced simulator, which is composed of seven high-definition projectors that provide 300 FOV to drivers [82]. In terms of vertical FOV, the highest value is found in [93] due to the use of large screens surrounding the simulator.

The simulated scenarios contain various types of roads (urban, rural, highway, single lane, and multilane), with lengths varying from 1 to 38.6 km. The lengths were reported by the authors in either kilometers, meters, miles, or feet but were transformed into kilometers in this paper. The longest route is presented in [94], having 24 miles (equivalent to 38.6 km). As for the duration of the experiments, it varies from 2 min [33] to 120 min [63,95]. In this case, only 40 of the articles reported the duration of the experiment.

Fourteen studies reported that the simulator uses an automatic transmission, seven studies stated that a manual transmission was used in the experiments, and the rest of the papers did not explicitly state this information.

The impact of the secondary task was assessed in various driving scenarios. Of these, two types were identified as the majority: 19% of studies (*n* = 13) used a car-following scenario, which requires following a lead vehicle and responding to its behavior [96] and which is the most common routine driving situation [97]. In 50 studies (75% of the total number of articles), the first task was to free drive on a route or to follow a path along which one or more incidents occurred. Examples of such incidents include the sudden appearance of an animal on the roadway [29,81], the sudden appearance of a pedestrian crossing the street [18,19,20,51,60,65,76,90], a cyclist entering the roadway [36,51,65], a parked car pulls out onto the road [18,90], and so on. 

Apart from car-following and free-driving scenarios, the other articles contain the following scenarios: a crossing road [88], rail level crossing [31], steering along the lane’s center [87], and lane changing [98].

#### 3.3.2. Driver-Tracking Equipment

The information about the driver’s performance was collected through the hardware and software systems of the simulator, but in 33% of the total number of studies, additional driver-tracking devices were used. Thus, in twenty articles, a device for tracking the driver’s gaze was used; in one article, brain–computer interface (BCI) systems were used; and in one article, the whole body of the user was tracked. For eye-tracking, some researchers used simple video cameras and extracted the information by manual coding of the recorded video: [54,58,60,68,93,99]. Others used specialized eye-tracking devices: Fovio eye tracker [20]; Ergoneers’ Dikablis Essential head-mounted eye tracker [36,55]; eye-tracking system developed by Seeing Machines, Ltd. (Canberra, Australia): faceLAB™ 4.1 [90]; faceLAB™ 5.0 [31]; Pupil Lab’s Pro head-mounted eye tracker [100]; SmartEye6.0 [69]; eye-tracking glasses developed by SensoMotoric Instruments, Berlin, Germany [74,78,101]; Tobii Pro Glasses 2 [80,84], Ergoneers Dikablis Eye Tracker 3 glasses [102]; and one paper did not mention the device. A MindCap XL headband equipped with a NeuroSky sensor was used to measure brain activity [59]. In [33], a high-speed infrared camera Motion Analysis Corp., Santa Rosa, CA, USA, was used to track the full body of the participants.

Four papers considered the physiological data taken from the participants during the experiment. In these studies, heart rate and skin conductance were measured using devices such as the MEDAC System/3 instrumentation unit by NeuroDyne Medical Corporation [54,68] and Biopac BioNomadix3 MP150WSW system [60], and heart rate plus other cardiovascular reactivity indicators (root mean square of successive differences, systolic blood pressure, diastolic blood pressure, and mean arterial pressure) were measured in [65].

### 3.4. RQ3: What Measures Were Used to Analyze and Predict Distraction?

**Figure 7:**
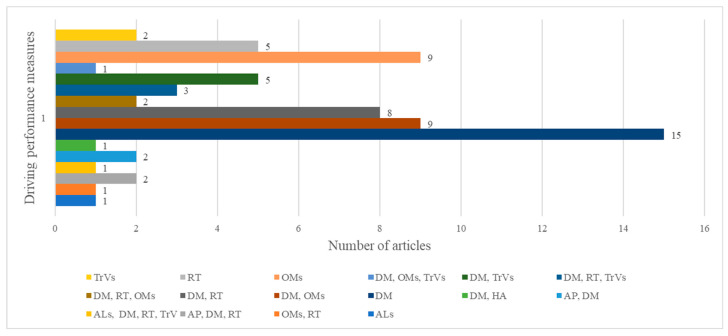
Distribution of papers according to driving performance measure categories (TrVs—traffic violations; DM—driving maintenance; ALs—attention lapses; RT—response time; HA—hazard anticipation; AP—accident probability; and OMs—other measures).

The selected studies include several measures to assess driving distractions. Most of them are driving-simulator-dependent variables used to assess the driver’s performance under the influence of distractions. Choosing such measures is an appropriate approach in the context of car simulators, as no additional sensors are needed. We grouped driving-performance measures into seven categories, starting from the classifications found in [103] and [104] and adding a new category regarding variables that are not necessarily related to vehicle-performance parameters: traffic violations (TrVs), driving maintenance (DM), attention lapses (ALs), response time (RT), hazard anticipation (HA), accident probability (AP), other measures (OMs). The distribution of papers according to these categories is presented in Figure 7. In some studies, variables belonging to only one category are used, while in others, they are part of two, three, or even all four categories. Most articles used measures from the DM category (49 studies), followed by RT (22 studies), OMs (21 studies), TrVs (12 studies), AP (4 studies), ALs (2 studies), and HA (1 study).

In the DM category, the following measures were included: lane-keeping measured by the standard deviation of lateral position (SDLP) [35,60]; speed variables, such as mean speed [19,34,105] and standard deviation (SD) of speed [34]; steering control, including steering angle [106,107] and SD of steering angle [17]; time to collision [64]; and headway measured in space–distance headway [88] or in time–time headway [108].

**Figure 8:**
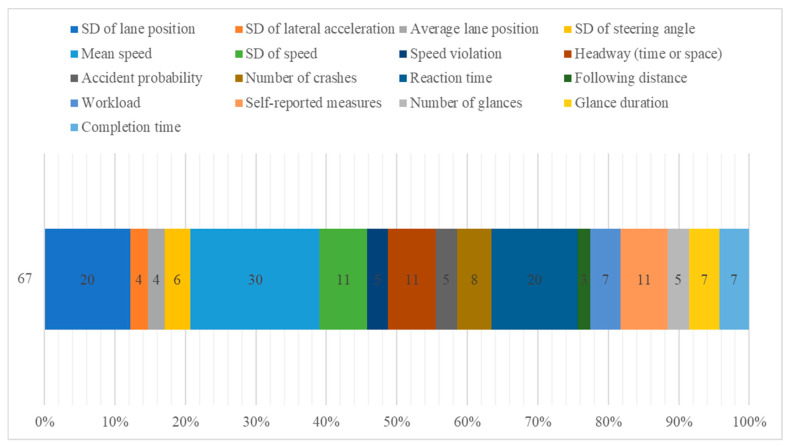
Main measures used in the experiments of the examined studies (SD—standard deviation).

RT includes brake reaction time [20,109] and other time variables in response to a pop-up event [18]. In the TrVs category, variables such as speed violation [72] and the number of collisions [77] were considered. ALs include results related to cognitively demanding and texting compared to four different blood-alcohol-concentration (BAC) levels: 0.00, 0.04, 0.07, and 0.10 [85]. OMs consist of other variables that cannot be included in the categories presented above: task completion time [67,68]; workload [87]; or variables related to eye tracking, such as the number of glances [78,84], off-road glances [54,69], and saccade amplitude [102]. The most common measures that were examined in the analyzed studies are presented in Figure 8.

In addition to measures related to the driving performance or other types of outcomes measured using sensors or self-reported, some of the studies also took into account additional parameters or independent parameters, such as the age of participants (A), driving experience (E), gender of participants (G), weather (W), road configuration (RC), and traffic flow (T). There are 18 articles that analyzed these additional parameters. In most studies, age was considered to be an independent parameter (11 studies), followed by gender (3 studies), driving experience (3 studies), traffic flow (2 studies), road configuration (2 studies), and weather (2 studies). There are studies that consider two or more parameters: A and E [32,100], A and G [19,86], RC and T [18], and RC and W [91]. 

Related to the statistical analysis of data, the most used technique was the analysis of variance (ANOVA), being applied in 33 of the selected studies. Other statistical methods used in the works were multivariate analysis of variance (MANOVA; 1 study), Wilcoxon signed rank test (10 studies), Wald test (6 studies), t-test (8 studies), regression analysis (3 studies), logistic regression analysis (1 study), linear mixed models (2 studies), and generalized linear model (2 studies).

### 3.5. RQ4: What Is the Impact of Using Mobile Devices to Read and Write Messages While Driving?

The selected studies were found to vary in several aspects: the proposed objective, the number of participants in the experiments, the infrastructure used to pursue the proposed objective, the outcomes, and so on. However, there is an agreement between the main outcomes of these studies. That is that text messaging, which mostly involves visual and manual distraction, has a significantly larger influence on driving performance [66] than a phone conversation. The main effects of this secondary task are increased variability in lane position and missed lane changes [90], increased brake reaction time [82], greater speed variability [110], increased steering variation per second [30], and higher completion times [88], as well as a higher risk of accidents than other in-vehicle tasks, such as tuning the car radio [67]. Even though drivers are aware that it is dangerous [98] and illegal in many countries to use a mobile phone while driving, they cannot resist the temptation to read and reply to messages, especially in the case of younger drivers [64]. Sending or reading a text from a smartphone takes the driver’s eyes off the road for 5 s, and, at a speed of 55 mph, that is similar to driving the length of an entire football field with the eyes closed [111].

Another secondary activity that has a negative impact on the driver’s performance is using social media [63]. However, this was not found to be as detrimental as texting [20] since image-based interfaces may provide a safer way to stay connected while driving than text-based interfaces [82]. Moreover, the side effects of using social media can be prevented with the help of advanced driver-assistance systems (ADASs) [80].

Visual–manual distractions negatively influence lateral lane position variability [112] and the average speed [57] by taking the driver’s eyes off the road [58] and increasing the mental workload [78]. Auditory distraction has been studied less, but it also seems to affect drivers’ performance by negatively affecting situation awareness and mean speed [113]. However, driving performance is less affected when travel information is presented in auditory mode [93]. A proper user interface (UI) design of smartphone applications could reduce the visual and cognitive demands of the driver when engaged in secondary activities. However, there is plenty of room for improvement of UIs in the automotive context. One design feature that could alleviate the drivers’ visual–manual demands is the integration of speech-to-text technology in either mobile phones or in-vehicle systems [55].

Using a mobile phone while driving can lead to compensatory measures to mitigate the effect of the distraction. Drivers could increase their vigilance [106], adopt a reduced speed [19,67], increase their distance from the leading vehicle [114], and self-regulate the secondary task [112]. It is worth noting that the driving task also negatively influences the texting task by inducing accuracy errors [115] and an increased response [116].

Regarding the independent variables, some findings can be extracted from the analyzed studies. The driver’s age can be used to predict driving performance significantly when it is correlated with the driving experience. To illustrate this aspect, [72] found that teens are not responsible enough while driving, as they have inadequate vehicle-control abilities and are more likely to be distracted from HH phone tasks compared to older drivers. However, young people have lower longitudinal control during distracted driving [32] and are more likely to accept a gap in intersections [88]. The age may be counterbalanced by driving experience, but in the case of TWD, it does not have any influence. In terms of gender, it was found that male drivers drove at higher speeds [19], while female drivers performed a higher number of lane excursions and had a higher reaction time compared to male drivers [17,18,75]. Moreover, male drivers tend to be more positive toward on-board traffic messages and in-vehicle systems [86].

Regarding the road configuration variable, it was observed that road geometry (especially curved road and vertical alignments) has a more significant influence on speed and lateral position than mobile-phone distraction [89]. Furthermore, it was found that text messaging could lead to behaviors that can obstruct traffic flow [94]. 

Another relevant outcome is that weather does not seem to influence the mean speed, but it can negatively affect the mean reaction time [91]. 

Some secondary tasks, such as eating and drinking while driving, have fewer distracting effects on the driver’s performance than phone texting [29,56]. In addition, operating a music player was found to be less risky than texting, which was reported to be an extremely risky task [71]. Studies that analyzed drivers’ physiological data showed that TWD increases cardiovascular reactivity [65] and skin conductance [68] compared to driving with no secondary tasks.

Several studies that explore the impact of texting on driving behavior have shown that engagement in secondary tasks directly influences safe driving performance [33]. For instance, regardless of the device, whether it is a mobile phone or a smartwatch, if the driver’s gaze is not on the road scene and all attention is on the device and its contents, then the driving performance is affected [68,78], and this, in turn, increases the risk of a crash [36]. The probability of a crash increases up to four times when drivers are engaged in distractions related to using a mobile phone [19]. The use of augmented-reality glasses did not eliminate the distracting cognitive demands while driving and still influenced driving performance [54]. The age of the participants is the main limitation of the analyzed studies, which included the use of Google Glass, as they include mainly a younger segment of the population. A summary of the results of the selected papers can be found in Appendix A Table A1.

## 4. Discussion

The primary focus of this comprehensive review is to summarize the existing knowledge regarding the impact of texting and reading on a mobile phone while driving in a simulator. The review addressed four research questions that can help to better understand the distractions that influence the drivers’ performance, what simulators were used by researchers, and what measures were considered to assess the impact of distracted driving. The review found a relatively large number of studies (*n* = 67) that addressed texting as a secondary task while driving in a simulator. The results of the review are in line with those of previous research, which found that TWD has a negative effect on a number of parameters related to driving performance that can be investigated in experiments conducted in car simulators. 

The included studies can be divided into two broad categories depending on the device type: handheld or hands-free devices. The sources of distractions were also classified into the following four types: cognitive, visual, manual, and auditory. Most secondary tasks include at least two distractions that can influence the driver’s ability to reach his/her destination in a safe manner. The driver’s brain has to manage all of the abovementioned distractions when operating a vehicle. Any additional distractions can increase the mental workload, thus compromising the driver’s performance. 

Drivers are subject to various distractions that can hamper their driving ability. Manual and visual sources of distraction are the most common and correspond to activities such as interaction with in-vehicle devices or the use of a mobile phone. Driver-assistance systems that offer warnings could reduce the time the driver is not focused on the driving task. Some high-end vehicles already have integrated devices that track the driver’s gaze. However, technology needs to become more accessible, reliable, and mainstream. We expect to see rapid progress in deep learning algorithms that can accurately identify and track the driver’s gaze by using a simple video camera. 

The driver’s behavior has been exhaustively researched in naturalistic and simulator-based studies [117,118]. Even so, there is still work to be performed to fully understand the combination of measures most effective in predicting road safety. The most popular variables used by researchers to analyze driving patterns are mean speed, reaction time, and the standard deviation of the lane position. 

Driving scenarios investigating hazard anticipation and traffic violation measures in a simulator are gaining more and more interest. The negative effects of using a mobile phone for TWD have been confirmed by numerous studies. The main effects include an increased brake reaction time, a decrement in lane control, and higher speed variability.

### 4.1. Recommendations and Directions for Future Research

What is evident from the findings is that typing and reading text messages while driving, regardless of the device used, should be prohibited in order to reduce the number of traffic-related deaths and injuries. Although it is advisable not to use a phone while driving, this is not very likely to happen, as it is used for various purposes, and the tendency to check the smartphone’s screen cannot be easily inhibited [119]. To support this idea, it was shown that even the experience of a minor accident is not enough to discourage drivers from sending messages while driving [120]. A possible solution would be to reduce as much as possible the unnecessary use of the phone and provide easy access to its screen by placing it in the field of view of the driver in a way that he/she is still attentive to the traffic scene or by sharing the screen on built-in display systems, which should be safer to use while driving. Moreover, built-in driver-assistance systems that prevent distracted driving should become mainstream as soon as possible, especially considering the rising number of traffic participants involved in car crashes due to phone use. A solution that has been shown to be effective would be the intervention by interactive text message [121].

A topic that still requires attention is how to increase the use of advanced driving-assistance systems (ADAS) to prevent drivers from engaging in distracting secondary tasks. For instance, ADAS systems may reduce or prevent the excessive use of a mobile phone by giving visual–audio notifications when the driver takes his/her eyes off the road. Future studies should focus on reducing the number of false alerts and propose adaptive ADAS models that can modify their behavior according to the characteristics of a driver (some initial work is presented in [122]). The use of safety functions should not impose other costs, as most drivers would not pay extra for such systems [123]. Another key aspect that could increase the acceptance of ADAS is related to the education of the driver, which should fully understand the safety benefits and limitations of such systems.

After analyzing the included studies, we noticed a lack of consensus regarding the methods and materials used for running experiments in driving simulators. In the context of automation, we suggest some minimum features for DS to ensure high reliability, validity, and replicability of the obtained results. The need for a systematic comparison of DSs concerning their validity and fidelity was also expressed in a scientometric analysis in [124]. Other issues identified are related to simulation sickness, how drivers perceive risks in a virtual environment, and the lack of detailed descriptions in research studies. A DS that offers high validity has the ability to reproduce as accurately as possible real-world driving [125], but the validity should be investigated in-depth to better approach the real conditions of driving [126].

Several aspects need to be considered when testing whether a driving simulator provides valid results: the simulator itself, the user samples, the task studied, the design of the experiment, and even the terminology used [34]. In view of these, and given that car manufacturers, taking advantage of the latest technologies, are setting new standards for car simulators [127], we propose several recommendations for future research in the context of driving simulators (the summary is shown in Table 1):

- Hardware characteristics: The simulator should have a dashboard resembling that of a real car, providing at least three DOFs in terms of motion and having a display system that offers a minimum horizontal field of view of 135° [128]. It should have the basic vehicle controls, a sound system, and at least a system capable of monitoring the driver’s behavior, which includes functions that can detect distracted driving. Distraction-detection systems are important in the case of autonomous driving because automated-vehicle drivers will still need to be in the loop in order to take over the controls when necessary [129].
- Scenario—Driving scenarios should provide a similar experience to naturalistic driving [130] and highlight the different types of driving behavior [131]. Therefore, we consider that it is not enough to consider a single basic scenario and suggest that experiments should include at least two driving situations, having multiple driving conditions (for example, driving in urban, rural areas, less or more traffic, simpler or more complex road geometry, etc.).

The driving task should not be too long in order to avoid fatigue and boredom, but not too short in order to be able to extract relevant results. Participants need to be monitored in case they experience simulator sickness during the practice session and in the study itself. A subjective evaluation of the experiment, for example, using questionnaires to better understand how the experiment influenced the driver’s psychological state (e.g., discomfort, fatigue, workload, frustration, mind wandering, and so on), can be beneficial and generate other valuable insights.

Therefore, punctual research studies that focus on a particular subject or concern are frequently carried out over a shorter period and might utilize a smaller sample size and a limited number of techniques to gather data. These studies might also look at the efficacy of measures taken to reduce the harmful effects caused by particular driving distractions. On the other hand, in order to gain a thorough understanding of a specific topic, it is crucial to gather a large amount of data over time and under different driving conditions, which, in turn, can reveal significant trends and patterns.

### 4.2. Limitations

Certain limitations need to be mentioned for this review. First, since the use of the mobile phone while driving is a widely studied field of research, it is possible that some relevant articles may have been missed even after a rigorous search of the literature. The review was limited to excluding studies published in conference proceedings or book chapters, as well as those published in languages other than English. Some shortcomings are related to the data, which were not fully reported in several papers. There are also methodological limitations, including the lack of valid and reliable measures to assess the effects of TWD, the use of small samples, the duration of experiments, and so on.

The proposed recommendations aim to offer guidelines for experiments using a driving simulator. However, they cannot consider all the possible scenarios that could be investigated. The suggested minimum requirements are based on the knowledge gained from the literature review analysis and on our partially subjective vision of driving simulators. It can be argued that a consensus regarding this topic will, perhaps, never be reached, as researchers will just use the infrastructure available. 

## 5. Conclusions

This study presents the results of a review of the literature using a structured search to examine drivers’ use of mobile phones and wearable devices concerning simulated driving. Through a rigorous selection process, fifty-nine studies published in the past 20 years were extracted, analyzed, and classified into four categories. Advanced driving simulators with a motion system were used in less than 20% of the studies due to the high costs and complexity of operation and maintenance. According to [132], studies that include low-cost simulators to identify and analyze the driver’s performance can offer meaningful and even similar findings as those obtained from experiments with advanced driving simulators. Nonetheless, the lack of a motion platform significantly affects the realism of the simulated scenario, as the participant cannot experience the vehicle’s inertia when accelerating or when negotiating a curve. 

Mobile phone use in the vehicle is a major component of distracted driving that requires drivers to take their eyes off the road and one or both hands off the steering wheel, thus impairing their driving performance and increasing the likelihood of crashes [133]. Most studies reached the conclusion that activities such as texting a message on the phone, manipulating the phone, or the use of different types of phone-connected devices can introduce cognitive, manual, visual, or even auditory distractions [134] that can have serious negative effects on drivers’ attention and concentration, and this can lead to serious traffic incidents [135]. 

Many studies based on driving simulators show that performing secondary tasks (such as manual input) while driving leads to a compromised driving performance [17,18,19,32,70,101,136]. Distraction can be achieved by removing the driver’s gaze from the road. However, cognitive distractions can be just as dangerous by taking his/her mind away from the driving process [137]. 

The ubiquity of mobile phones; the increasing number of traffic participants; and their need/desire to engage in secondary tasks, such as games, texting, or social media, could have a negative effect on road safety, despite the integrated or mobile driver assistance systems. This review can serve as a basis for regulators and interested parties to propose restrictions related to using mobile phones in a vehicle and improve road safety. It also points out the significance of informing drivers about the dangers of using mobile phones while driving and the importance of enforcing strict rules and sanctions for those who have a habit of doing this. Moreover, the study provides researchers with an overview of the types of distractions that can affect the driver at a cognitive, visual, manual, or auditory level, as well as the measures that can be used to predict and analyze those distractions. The review recommends that future research should concentrate on creating more sophisticated driver assistance systems and technologies that can better detect and prevent distractions caused by TWD.

Future research should focus on finding a consensus regarding driving-simulator studies that will enable scholars to directly compare their work with similar studies, thus ensuring high validity of results, especially in the context of automated driving. 

## Figures and Tables

**Table 1:** Minimum feature recommendations for experiments using a driving simulator.

	Immersion:		
	Motion Platform	Display	Other Features
Hardware features	3 DOFs	At least 135° horizontal FOV and 40° vertical FOV	- Dashboard similar to that of a real car - Basic vehicle controls - Sound system
	Driver tracking:		
	Movement	Distraction detection	Physiological metrics
	Head tracking	Eye and/or hand tracking	Electrocardiogram (ECG) 10^3^ A/m
	Number	Type (difficulty)	Driving conditions
Scenarios	Minimum 2 scenarios, including a baseline	- urban/rural environment - Low/ heavy traffic - Curves, hills, intersections, and roundabouts	- Day/night - Rain/snow/sun/fog

## Data Availability

Not applicable.

## Appendix A

**Table A1:** An overview of driving simulators characteristics and classification (*n* = 67).

ID	Ref.	NP	Sample Characteristics ^a^	Driving Simulator Class ^b^	LSR (km)	TD	MT	Type of Device—Distraction Task	Findings
1	[70]	35	NR; 22.5; NR; 21–14	B	2,65	V, C, M	TrVs, DM	HH—texting	Based on vehicle dynamics, it is possible to identify specific distraction tasks with a level of accuracy that is adequate.
2	[54]	25	22–33; 25; 2.6; NR	A	NR	V, M, C	OMs	HF—destination entry	In comparison to the primary visual-manual interaction with the Samsung Touch interface, voice entry (from Google Glass and Samsung) resulted in lower subjective workload ratings, lower standard deviation of lateral lane position, shorter task durations, faster remote Detection Response Task (DRT) reaction times, lower DRT miss rates, and less time looking off-road.
3	[50]	134	20–30, 65–75; 23.2, 70.0; 2.8, 3.0; 23–40, 39–22	A	25.7	V, Au + A	DM	HF—typing a number into a keypad, conversation with a car passenger, memorizing	Braking responses are affected by distractions, and this effect can last for up to 11.5 s.
4	[78]	31	18–47; 25.61; 6.24; 16–15	A	NR	V, C, M	TrVs	HH—received and answered text messages	Any mobile gadget, like a smartwatch, smartphone, or voice assistant, could affect how well you drive, especially if you have to pay attention to it when your eyes are off the road.
5	[93]	24	NR; 33, 26.3; NR; 8–4, 8–4	B	NR	V, C, Au	DM	HF—receives traffic information	The two other systems required the participants to glance away from the road (too) long, endangering their safety, and reading an SMS took longer than scanning a PDA. The auditory information provision system, however, provided for the best driving performance.
6	[83]	39	19–32; 21.5; 2.6; 27–12	A	NR	V, C, M	TrVs	HF—respond to a call, replay several WhatsApp messages, use Instagram	Young drivers who use mobile phones while operating a vehicle experience impairments that limit their ability to control the vehicle.
7	[108]	53	22–34; 25.25; 3.08; 37–16	B	3	V, C, M	RT	HH—speech-based texting and handheld texting (two difficulty levels in each task)	Drivers undertake risk-compensation behavior by extending time headway in order to offset the higher accident risk associated with using a mobile phone while driving. Drivers perceive a rise in accident risk during distracted driving.
8	[102]	41	<25, 26–40, >41; NR; NR; 30–11	B	20	V, M + A	DM, OMs	HF—enter the application interface of 3, 4, or 6 icons	In the HMI design of in-vehicle information, there is a statistically significant difference in driver perception reaction time for varying numbers of icons (IVI).
9	[17]	100	<30, 30–50, >50; 24.14, 36.05; 54.67; 2.79, 5.43, 5.04; 87–13	B	3.5	V, C	DM	HH—simple conversation, complex conversation, and simple-texting and complex-texting tasks	Both talking on the phone and texting while driving impair a driver’s ability to pay enough attention to the road ahead, to react appropriately to unexpected traffic situations, and to control the car within a lane and in relation to other vehicles.
10	[18]	100	<30, 30–50, >51; 24.14, 36.05, 54.68; 2.79, 5.43, 5.05; 87–13	B	3.5	V, C + RC, T	RT	HH—simple conversation, complex conversation, and simple-texting and complex-texting tasks	Simple conversations, complicated conversations, basic texts, and complex texts all increased reaction times for pedestrian crossing events by 40%, 95%, 137%, and 204%, respectively. For parked car crossing events, the tasks increased reaction times by 48%, 65%, 121%, and 171%, respectively.
11	[19]	100	<30, 30–50, >52; 24.14, 36.05, 54.69; 2.79, 5.43, 5.06; 87–13	B	3.5	V, C + A, G	DM, AP	HH—simple conversation, complex conversation, simple texting and complex texting tasks	When engaged in conversation or texting duties, the drivers significantly decreased their mean speed by 2.62 m/s and 5.29 m/s, respectively, to offset the increased strain.
12	[32]	49	22.12, 37.62; 22.12, 37.62; 2.45, 7.22; 22–3, 25–0	B	3.5	V, C + A, E	DM	HH—simple conversation, complex conversation, simple texting and complex texting tasks	Younger drivers are less able to compensate for distractions while driving and have poorer longitudinal control.
13	[71]	90	<30, 30–55; 25.31, 37.00; 2.74, 6.29; 83–7	B	NR	V, M + A	DM, RT	HH—conversation, texting, eating, music player	Most of the drivers (72.06%) reported texting as an extremely risky task
14	[49]	14	18–22; NR; NR;	B	NR	C, M	DM	HH—cell phone conversation, back seat conversation, text message, Ipod manipulation	The iPod task and all wireless communication tasks caused a noticeable increase in speed variability throughout the driving scenario.
15	[86]	49	19–65; 35.63; 14.26; 32–17	B	50	V, C + A, G	OMs	HH—reading and comprehension task (three types of display)	Warnings took longer to read and comprehend (4 s on average), compared to recommendations.
16	[66]	40	19–23; 21; NR; 20–20	B	51.5	V, M	DM, RT	HH—text messaging	Simulated driving performance suffers when texting while operating a vehicle. This detrimental effect seems to be more severe than the consequences of using a cell phone for conversations while driving.
17	[80]	17	NR; 25.88; 5.82; 14,3	B	NR	V, M	TrVs, DM	HH—accessing social network on the smartphone	Even when the driver is distracted, using an in-vehicle smartphone ADAS application has enhanced driving performance in a simulator..
18	[56]	101	18–57; 27.8; 8.3; 68,33	A	NR	C, V, M	DM	HH—using a handheld cell phone; texting; eating	Regardless of their prior experience, multitasking while driving and distracting activities have a negative influence on driving performance for both genders and all age groups. The main factor that negatively affected driving performance was texting.
19	[109]	56	21–30; 25.13; 2.57; 41–15	B	3	V, C, M	RT	HF, HH—speech-based and handheld texting	Compared to the baseline, handheld texting tasks caused a delayed reaction to the unexpected braking occurrences.
20	[36]	26	22–31, 22–29; 25.5, 23.9; 3.33, 2.27; 3–3, 20–0	B	NR	V, M + A	RT, DM	HH—receive notification	The use of smartwatches could affect traffic safety. There may be a discrepancy between drivers’ actual performance and their views regarding using a wristwatch while driving, given that participants generally believed that smartwatch use resulted in similar or fewer traffic fines than smartphone use.
21	[55]	48	20–79, 19–66; 34.8, 35.3; 16.0, 13.9; 17–7, 16–8	C	NR	V	OMs	HH—email reading, view-switching, song searching, email replying	Compared to using standard smartphone apps, an automotive-specific application reduced the visual demand and visual distraction potential of in-car duties.
22	[72]	63	25–66, 8–18; NR; NR; 32–31	D	NR	V, M + A	DM	HH, HF—answer incoming calls, dialing, retrieve a voicemail message from a specific person using either the handheld or hands-free phone	Teenagers were shown to adopt risky following distances, to drive poorly, and to be more easily distracted by handheld phone tasks than adults.
23	[20]	36	NR; 20.95; 2.36; 16,10	C	6.8	V, C, M	RT, DM	HH—social media browsing	Performance is impacted by both texting and using social media, but texting while driving is more harmful.
24	[90]	20	18–21; NR; NR; 12,8	C	8	V, M	DM, HA	HH—retrieve and send text messages	Text messaging has negative consequences on driving ability, which could explain the higher crash risks.
25	[99]	24	18–64; 32.1; 12.5; 10,14	A	3.55	V, M	DM	HH—manual dialing, voice-dialing	When participants utilized voice-activated dialing as opposed to manual dialing, there were 22% fewer lane-keeping mistakes and 56% fewer looks away from the road scene.
26	[69]	40	20–52; 32.5; NR; 11,29	B	NR	V, C	OMs	HH—touching the touch-screen telephone menu to a certain song, talking with laboratory assistant, answering a telephone via Bluetooth headset, and finding the navigation system from Ipad4 compute	The attention of the driver is substantially diverted from the road when engaging in secondary tasks while driving, and the evaluation model used in this study could accurately predict driving safety under various driving circumstances.
27	[61]	24	20–45; 33.43; 6.32; 22–2	A	NR	V	DM, RT	HF—ordering, route check, destination search	Usability and driving safety were higher when the phone was placed on the left side of the steering wheel as opposed to the right.
28	[33]	29	NR; 56.6, 55.9; 4.1, 3.0; 16, 13	A	NR	V, M, N	RT, OMs	HH—sending a text message, searching navigation	When driving while sending a text message or using navigation, the jerk-cost function, medial-lateral coefficient of variation, and braking time were all higher than when driving alone.
29	[58]	20	27–59; 37.65; 9.75; 14,6	B	10 + 9	V, M, C	DM, OMs	HH—conversation, texting, destination entry, following route guidance	Only when individuals engaged in visual-manual tasks, such as texting and entering a location, when they frequently glanced away from the forward road, did lateral performance decline.
30	[64]	30	18–30; 22.7; 3.51; 15,15	A	13	C, M	DM, TrVs	HH—“temptation to text”	The “Temptation to Text” condition revealed noticeably more workload. Similarly, it was discovered that texting while driving drastically reduced vehicle performance.
31	[85]	20	23–30; 26.20; 2.58; 10,10	A	NR	C, M	TrVs, DM, ALs, RT	HF—conversation, HF cognitive demanding conversation, texting	Comparatively to legal BAC limits, very basic mobile phone conversations may not pose a substantial risk to driving, but cognitively taxing hands-free talks and, most notably, texting, do pose significant dangers.
32	[88]	41	18-61; 31; 9.7; 23,18	B	5	C + G	ALs	HF, HH—conversation	Drivers’ decisions regarding accepting gaps were unaffected by the distraction task, although the crossing’s completion time increased by over 10% in comparison to the baseline. Also, when using a phone at an intersection, drivers exhibited conservative behavior, slowing down more quickly, waiting longer, and keeping a greater distance from the vehicle in front of them.
33	[101]	29	22–49; 30; 6; 15,14	A	1	V, M	DM	HH—help, browse, filter task	The filtering task’s slider widget was overly demanding and hindered performance, whereas kinetic scrolling produced an equal amount of visual distraction although requiring less precise finger pointing.
34	[59]	15	NR; 28; 4.08; 12,3	A	NR	C, V, M	OMs	HH—button, slider, Insert data, dropdown, radio buttons	When evaluating the mental workload related to wide differences in task complexity in terms of the amount of information to be processed, a commercial BCI device may be helpful.
35	[75]	60	16–17; 16.8; 0.4; 20, 40	B	NR	V, M + G	OMs	HH—looking at the phone, picking up the phone, taking a picture, sending the picture, hand manipulation of phone (mimicking writing a text), answering a call, and looking at a picture on the phone	Self-reported distracted driving habits grew with time, with a significant effect of visit on self-report outcomes.
36	[67]	28	18–28; 21.0, 2.4; _; 16,12	B	1.1–1.5	V, M	DM	HH—type and send a text message vs,. tunning car radio	Even in the simplest of driving situations, multitasking while operating a motor vehicle can have a negative impact on performance and increase risk. Comparing text messaging to other in-car activities like changing the radio, text messaging may present a “perfect storm” of risks.
37	[82]	18	18–22; 20.4; NR; NR	C	NR	V, M	RT	HH—text messaging, reading Facebook posts (text/self-paced), exchanging photos via Snapchat, and viewing updates on Instagram	When compared to the image-based scenario (mean = 0.92 s) and the baseline, the brake reaction times (BRTs) in the text-based scenarios were substantially longer (mean = 1.16 s) (0.88 s). Both the task-pacing impact and the difference between BRTs in the image-based and baseline conditions were not statistically significant.
38	[63]	64	22–60; 33; 10; 34, 30	D	NR	V, C	RT	HH—reading, texting, video, social media, gaming, phoning, music	Reaction times did decrease when performing non-driving related tasks (NDRTs), suggesting that the NDRT assisted the drivers in keeping their focus during the partially automated drive. Drowsiness and the NDRT’s motivational appeal thus raised situation criticality, whereas the NDRT’s cognitive load decreased it.
39	[89]	35	18–29; 22.9; 4.0; 22, 13	D	10	V, M, C + RC	DM	HF, HH—calling, texting vs. road environment	Compared to distraction from a cell phone or other road elements like pedestrians and approaching vehicles, road geometry has a greater impact on driver behavior.
40	[76]	35	18–29; 22.9; 4.0; 22, 13	D	NR	V, M, C	OMs	HH—ring a doctor and cancel an appointment, text a friend and tell him/her that the participant will be arriving 10 min late, share the doctor’s phone number with a friend, and take a ‘selfie	The three types of self-regulation that distracted drivers use most frequently are tactical, operational, and strategic.
41	[30]	50	27–55; 36.8; 5.8; 50,0	D	NR	V, M, C	DM	HH—driving while having a conversation on the mobile phone, driving while reading out loud text messages and driving while texting	The “reading of text messages” and “texting” had a big impact on the “change of the steering position per second. For all three cell phone assignments, a substantial main effect was seen in terms of “following distance per second” and “change of the lateral lane position per second”.
42	[29]	90	NR; NR; NR; 73,17	A	3.6	C, V	DM, RT, TrVs	HH—using the mobile phone, drinking and text messaging	The disruptive variables have a negative impact on road safety due to cognitive distraction and mobility limitation (e.g., longer response times and more errors), on the one hand, and have a bad impact on the environment and the economy (e.g., increased fuel consumption), on the other.
43	[105]	36	21–54; 33.3; 8.6; 21–15	B	4.8	V, Au	DM, RT	HF—features presented via a mobile phone mounted near the line of sight	The findings indicated that new features with the greatest levels of urgency and criticality, such as Emergency Vehicle Warning (EVW) and Emergency Electronic Brake Lights (EEBL), would improve safety and make it easier for emergency vehicles to reach their intervention site.
44	[68]	36	NR; NR; NR; 18,18	A	NR	V, C, M, Au	RT, DM, OMs	HH—smartwatch vs. smartphone calling	By using a phone instead of just driving, participants shown increased off-road visual attention.
45	[73]	32	17–21; 19.0, 19.3; NR; 7,9	B	NR	V, M	DM, TrVs, RT	HH—manipulating controls of a radio/tape deck and dialing a handheld cellular phone	The time spent on tasks was marginally longer for participants who anticipated dangers compared to those who did not, but the difference was stable across tasks.
46	[87]	45	NR; 62.8, 24.3; 7.2, 4.8; 30–0, 11–4	B	NR	V, P	DM, OMs	HH—texting on a smartphone and while sitting on a stable or unstable surface	When drivers were texting, the perceived workload increased, but balancing training decreased it. While seated on the unsteady surface, perceived workload was higher; however, it decreased after balance training.
47	[35]	40	NR; 20.47; 4.76; 24, 16	B	8.04	V, M	DM, RT	HH—use Google Glass or a smartphone-based messaging interface	Glass-delivered messages served to reduce distracting cognitive demands, but they did not completely remove them. Comparatively speaking to driving when not multitasking, messaging while using either gadget impairs driving.
48	[81]	37	18–33; 24.7; 3.6; 20–17	B	NR	V	DM, RT, AP	HF—navigating on the Facebook newsfeed, reading and sending text messages in Facebook Messenger, searching for a location in Google Maps	Web browsing and texting-related distraction raise the likelihood of an accident, the headway, and the lateral distance deviation by 32%, 27%, and 6%, respectively.
49	[84]	123	18–64; 34.46; 13.04; 62,61	B	26.4	V, Au	DM, OMs	HH—audio warning, flashing display	There was no difference in the number of vehicles overtaken between the groups, and the existence of the speed warnings had no effect on overtaking.
50	[51]	34	16–18; 17.25, 17.09; 0.99, 0.89; 12–4, 14–4	B	8.04	C, M	DM, RT, TrVs	HH—conversing on a cell phone, text messaging	Compared to the no task and the cell-phone task, the lane position varied significantly more while texting. Teens with ADHD spent noticeably less time to finish the scenario while texting in particular. There were no discernible group-wide major effects detected.
51	[77]	50	24–54; 39.8; 8.4; 49, 1	B	36.2	C, M, V	TrVs, DM, OMs	HH—cell phone conversation, text message interaction, emailing interaction	Poorer driving performance was associated with more visually demanding jobs. Yet, using a cell phone caused fewer off-road eye looks. Drivers who described themselves as “extremely skilled” drove less well than those who described themselves as “talented.”
52	[94]	75	16–18, 19–25; 17.67, 23.39; 1.18, 1.81; 11–19, 23–22	B	38,6	C, M + T	TrVs, DM	HH—cell phone, texting	Texting generally resulted in more lane deviations and collisions. Text messaging was the most common form of distraction, which had a major negative influence on traffic flow. As a result, participants’ speeds fluctuated more, changed lanes less frequently, and took longer to finish the scenario.
53	[60]	32	18–25; 20.6; 2.1; 32–0	D	13	V	DM, TrVs	HH—gamified boredom intervention	The gamified boredom intervention promoted anticipatory driving while reducing risky coping strategies like speeding.
54	[132]	36	NR; 28.44; 9.26; 30,6	A	NR	C, V, M	DM	HH—conversation, texting	Driver performance in the longitudinal and lateral control of the vehicle for the texting event significantly declined during the texting task.
55	[113]	37	NR; 21; 3.63; 11,26	B	NR	C, Au	DM, OMs	HH—text-message distractions	For at least 10 s but no more than 30 s following the text message alert, situation awareness is negatively impacted. Participants’ mean speed increased during periods of distraction in the 10 s after receiving a mobile phone notification, which also resulted in a decrease in context awareness.
56	[100]	27	24–59; 42.4; 9.1; 11, 16	B	4.4	V, M + A, E	DM, OMs	HH vs. dashboard—texting with the smartphone in one hand (handheld drive) and texting while the phone is placed in a dashboard mount	Texting while driving when using a dashboard-mounted device impairs driving safety at least as much as texting while using a handheld device.
57	[98]	40	NR; 28; 12.6; 10,30	A	NR	V, M + E	DM	HH—texting	Mobile phone texting dramatically reduced the ability to drive. Driving experience had no bearing on the results, however highly skilled phone users’ texting use had a noticeably reduced negative impact.
58	[95]	40	NR; 18.6; 1.8; 11–29	B	NR	V, M, C	DM, OMs	HF, HH—conversation, texting, selecting a song	Although the amount of interference varied depending on the task, hands-free smartphone call created substantially less interference than texting and listening to music on an MP3 player.
59	[65]	60	NR; 19.74; 2.4; 30,3	A	8.04	C, M	OMs	HF—conversation, texting	Driving while texting was similar to driving while not doing anything. The results of this study highlight the need for further investigation into the long-term effects of secondary task use while driving on cardiovascular reactivity as well as the dangers of secondary task use while driving on the risk of cardiovascular disease or stroke.
60	[110]	36	18–56; 26.95; 5.076; 23,13	A	2.5	M	DM, RT	HH—cell-phone texting	Driver groups with phone-texting distractions exhibited larger speed variability, longer average following HWDs, considerably slower reaction times, and longer distances needed for quick recovery in response to front-car braking events than driver groups without such distractions.
61	[91]	34	18–28; NR; NR; 19,15	A	NR	V, M + RC, W	DM, RT, AP	HH—texting	In both urban and rural road contexts, texting results in a statistically significant decrease in mean speed and an increase in mean reaction time. Due to driver distraction and delayed response at the time of the incident, it also increases the likelihood of an accident.
62	[92]	34	18–24; NR; NR; 19,15	B	3	V, M + W	DM, AP	HH—navigation, tuning the radio, replying to a text message, replying to a voice message, and making a phone call	On highways, texting appears to cause drivers to exhibit compensatory behavior, which statistically significantly reduces the mean speed and increases headway in both normal and particular traffic and weather conditions.
63	[74]	34	NR; 47.6, 23.05; NR; 23, 11	A	NR	V, M + A	OMs	HF—normal conversation (non-emotional cellular conversation), and seven-level mathematical calculations	Making a call, returning a voicemail, and responding to texts are high-visual-load secondary chores that drivers shouldn’t engage in while operating a vehicle.
64	[62]	43	NR; 24.09; 3.27; 25–18	B	4.1	V, C	DM, OMs	HF—texting, talking	For basic road portions, texting considerably raised the SDLP, although conversational tasks showed less lateral variance than when there was no distraction.
65	[31]	28	18–55; 29.4; 11.3; 16, 12	B	9	V, M, Au	RT, DM, OMs	HH—text messaging	Although Glass enables drivers to better maintain their visual attention on the front scene, they are still unable to efficiently divide their cognitive attention between the Glass display and the road environment, which impairs their ability to drive.
66	[79]	20	22–47; 32.2; 6.3; 16, 4	A	3	V, C	DM, OMs	HH—reading text on Glass and on a smartphone	When approaching active urban rail level crossings (RLXs), texting had a negative effect on how well the driver performed.
67	[57]	101	18–57; 27.8; 8.3; 68, 33	A	6	V, C, M	DM	HH—texting, talking on the phone, or eating	According to the simulation results, texting and, to a lesser extent, talking on the phone cause traffic to move more slowly on average and with higher coefficients of variation.

Note: TD—type of distraction: C—cognitive, V—visual, M—manual, Au—auditory; MT—measure type: AL—attention lapses, AP—accident probability, DM—driving maintenance, HA- hazard anticipation, RT—response time, TrV—traffic violations, OM—other measures; HH—hand-held, HF—hands-free, NP—number of participants; LSR—length of simulated route; NR—not reported. ^a^ Values include age, mean, standard deviation, and gender (M, F). ^b^ Driving Simulator Classification: A—fixed-based, basic visual capability, FOV minimum H:40 and V:30; B—fixed-based, FOV minimum H:40, and V:30; C—motion platform, FOV minimum H:120 and V:30; D—minimum 6 DOF motion platform, FOV minimum H:180 and V:40 [40].
